# Emulating the EPIC trial using VetCompass primary-care data: causal effects of pimobendan in UK dogs with grade IV/VI heart murmurs

**DOI:** 10.1371/journal.pone.0325695

**Published:** 2025-06-18

**Authors:** Camilla Pegram, Karla Diaz-Ordaz, Dave C. Brodbelt, Yu-Mei Chang, Adrian Boswood, Jenny Wilshaw, Carmen A. T. Reep, Sarah Balling, Jaya Sahota, David B. Church, Dan G. O’Neill

**Affiliations:** 1 Pathobiology and Population Sciences, The Royal Veterinary College, Hatfield, United Kingdom; 2 University College London, Department of Statistical Science, London, United Kingdom; 3 Comparative Biomedical Sciences, The Royal Veterinary College, Hatfield, Herts, United Kingdom; 4 Clinical Science and Services, The Royal Veterinary College, Hatfield, United Kingdom; 5 Professional Services, The Royal Veterinary College, Royal College Street, London, United Kingdom; 6 Department of Intensive Care, Erasmus Medical Center, Rotterdam, the Netherlands; 7 Straid Veterinary Clinic, Beaconsfield, United Kingdom; King Saud University / Zagazig University, EGYPT

## Abstract

Target trial emulation applies design principles from randomised controlled trials (RCTs) to the analysis of observational data, potentially replicating RCT results in real-world settings. The EPIC trial reported that pimobendan delays the onset of congestive heart failure (CHF) and extends survival in dogs with preclinical degenerative mitral valve disease (DMVD). The current study aimed to explore the extent to which target trial emulation approximates the EPIC trial results in a primary-care setting. Grade IV/VI murmur diagnosis was defined as the treatment intervention stage.

There were 928 dogs ≥ 6 years and ≤ 15 kg at first grade IV/VI murmur diagnosis recorded from January 1, 2016, to December 31, 2018 in the VetCompass database included in the study. A causal inference “target trial emulation” approach using VetCompass anonymised clinical data was designed to replicate the EPIC trial with adaptation for a primary-care setting and to address immortal time bias, confounding bias and loss to follow-up.

After bias adjustments to establish causal effects using observational data, the 5-year CHF cumulative incidence was lower in dogs prescribed pimobendan (34.1%, 95% CI 26.5–42.0) than dogs not prescribed pimobendan (56.3%, 95% CI 52.8–59.8). Dogs prescribed pimobendan had 311 fewer days of health lost to CHF (95% CI 224–395 days) within 5 years. Dogs prescribed pimobendan lived longer (adjusted mean survival time 1051 days, 95% CI 967–1125) than dogs not prescribed pimobendan (905 days, 95% CI 871–940 days).

This study demonstrates that target trial emulation within veterinary research can replicate findings from RCTs. Clinically, the current findings suggest that preclinical grade IV murmur diagnosis may offer an appropriate intervention stage to begin pimobendan therapy in dogs with presumed DMVD.

## Introduction

Randomised controlled trials (RCTs) are considered the gold standard for assessing treatment efficacy but are not feasible or ethical for answering many therapeutic research questions [1,2]. “Target trial emulation” adapts RCT principles to observational data to estimate causal effects and is increasingly applied in human epidemiology [3]. Within this framework, observational studies are treated as conditionally randomised experiments that account for measured covariates [4–7]. Some successful applications of this approach have recently emerged in the veterinary literature [5–7] but no veterinary target trial has yet been designed around, or directly compared to, pre-existing RCTs.

The Evaluation of Pimobendan In Cardiomegaly (EPIC) trial was a multicenter, randomised, double-blinded, placebo-controlled trial that assessed the preclinical treatment of degenerative mitral valve disease (DMVD) in dogs [8]. The EPIC trial investigated the effect of pimobendan, a vasodilator and positive inotrope, on time to a composite primary endpoint of congestive heart failure (CHF), cardiac-related death, or euthanasia in dogs with stage B2 DMVD [8]. Based on the American College of Veterinary Internal Medicine (ACVIM) staging system, DMVD is classified into four categories (A to D), with stage B representing preclinical disease and C and D indicating clinical signs of CHF. Stage B is further subcategorised into stage B1 (no evidence of cardiac remodelling) and B2 (evidence of left atrial and ventricular enlargement) [9].

A recent study exploring whether non-echocardiographic factors could identify stage B2 DMVD reported that murmur intensity ≥ III/VI is associated with increased likelihood of stage B2 [10]. Murmurs are typically graded as I – VI, with grade I representing the least and grade VI the most intense murmurs [11]. Approximately 70% of dogs in stage B2 have a murmur of grade IV/VI or louder and 70% of dogs in stage B1 DMVD have a grade III/VI or quieter murmur [10]. However, more dogs are classified as stage B1 than stage B2, leading to a higher absolute number of grade IV murmurs in stage B1 DMVD [10,12]. Murmur assessment is a somewhat subjective measure, but can be detected during a routine physical examination and therefore facilitates effective diagnosis and management in dogs under primary veterinary care with DMVD prior to onset of CHF [10,11].

The EPIC trial demonstrated that pimobendan delayed CHF, cardiac-related death, or euthanasia by a median of 462 days and increased overall survival (median time to all-cause mortality 1059 days in treated dogs versus 902 days in untreated dogs in a 5-year follow-up period) [8]. The EPIC trial composite outcome choice can be justified as the outcomes are part of the same disease process [13]. Competing events, such as death from non-cardiac causes, can prevent CHF or cardiac-related death from occurring and have traditionally been censored in medical studies. However, this can result in upward biased estimates of the cumulative incidence of the outcome of interest [14]. More recently, appropriate competing events analysis, such as the cumulative incidence function, has been recommended [15].

Using anonymised primary care Electronic Health Record (EHR) data from the VetCompass Programme [16], the current study primarily set out to emulate aspects of the EPIC trial. Secondarily, the current study aimed to determine whether pimobendan prescription within 6 months of first detecting a grade IV/VI heart murmur in dogs leads to longer: a) time to CHF or cardiac-related death (considering death without evidence of being cardiac in origin as a competing event) and b) time to all-cause mortality, compared with no pimobendan prescription. Explicit diagnosis of stage B2 DMVD in primary-care practice is uncommon [17,18]. Therefore, first preclinical grade IV murmur diagnosis was identified as an appropriate intervention stage, since murmurs can readily be detected by primary-care practitioners during a routine physical examination and are associated with more advanced disease [19].

## Materials and methods

### Data source and power calculation

VetCompass collates de-identified electronic health record (EHR) data from primary-care veterinary practices in the UK for epidemiological research [16]. The DMVD study population included all available dogs under primary veterinary care at clinics participating in the VetCompass Programme during 2016. Dogs under veterinary care were defined as those with at least one EHR (free-text clinical note, treatment or bodyweight) recorded during 2016, or in both 2015 and 2017. Available data fields included a unique animal identifier along with species, breed, date of birth, sex, neuter status and insurance status, and also clinical information from free-form text clinical notes, bodyweight, summary diagnosis terms [20] and treatment with relevant dates.

Sample size calculations, using the ClinCalc Sample Size Calculator (www.clincalc.com), estimated that approximately 162 dogs per group were considered necessary to detect a difference in cumulative incidence of CHF of greater than or equal to 15% with a power of 80% and an alpha of 5%. Based on the EPIC trial findings, this assumes a 5-year incidence of CHF of 30% in dogs treated with pimobendan compared to 45% in dogs not treated [8]. Ethics approval was obtained from the RVC Social Science Ethical Review Board (reference number SR2018−1652).

### Case definition, case finding and covariates

The inclusion and exclusion criteria were designed around the criteria of the EPIC trial [8] with modifications for the observational primary care dataset to meet the information typically recorded in veterinary EHRs and so make the emulated trial more reflective of primary care practice. Eligibility criteria in the current study comprised dogs 6 years of age or older at first grade IV heart murmur diagnosis with a bodyweight ≤15 kg recorded in 2016 and first heart murmur of grade IV intensity recorded from January 1^st^ 2016 to December 31^st^ 2018. Dogs with evidence of cardiac-related clinical signs, congenital cardiac disease, cardiac disease other than DMVD and prescription of cardiovascular agents prior to first grade IV murmur diagnosis were excluded. In line with VetCompass methods used in several previous studies [5–7,18], candidate heart murmur cases were identified by applying search terms relevant to the diagnosis and management of DMVD in the clinical notes (including ‘grade IV’, ‘grade 4’,’B2’, pimo* and vetmedin). The search findings were merged, and a random subset of these dogs had their clinical notes examined manually in detail to identify dogs meeting the case definition.

**Fig 1:**
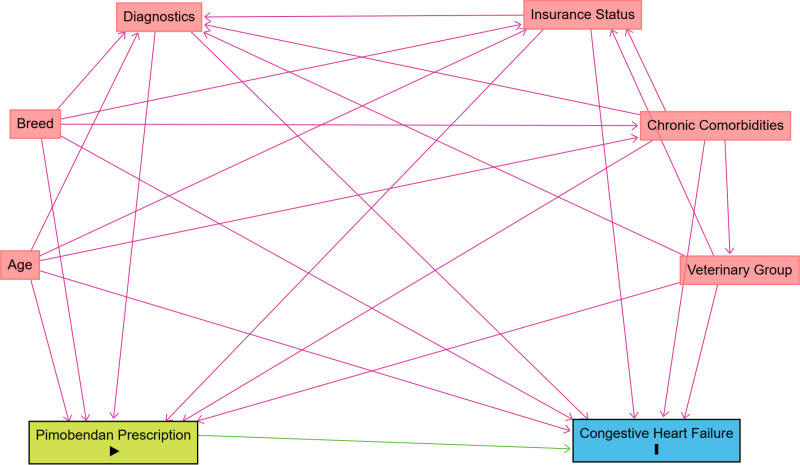
Directed acyclic graph (DAG), based on existing published evidence and expert knowledge, used to identify factors to control for when evaluating the effect of pimobendan prescription for degenerative mitral valve disease in dogs on congestive heart failure. The same causal structure was assumed to be sufficient to de-confound all-cause mortality as the outcome of interest.

Based on existing published evidence and expert knowledge, a directed acyclic graph (DAG) was constructed using DAGitty version 3.0, Fig 1, that encapsulated prior beliefs by the research team about the causal relationships relevant to the question of interest. The DAG was used to identify which variables should be controlled for in the modelling [21], and therefore data on the following baseline variables were extracted from the EHRs: age, breed, insurance status, chronic comorbidities, diagnostic tests performed and veterinary group (Fig 1). CHF and death were considered as outcomes in the following capacity: (i) CHF with death as a competing event and (ii) all-cause mortality. For simplicity, CHF only is referred to as the outcome (since no dogs in the current study had an unassisted death or euthanasia for cardiac-related reasons prior to onset of CHF), with the competing event of death. However, as in the EPIC trial, if cardiac-related death (unassisted or euthanasia) were reached without evidence of CHF, this could be considered as a composite outcome with CHF [8]. Based on existing evidence and expert opinion, the current study assumes that the same covariate sets are sufficient to de-confound both CHF (with death as a competing event) and all-cause mortality.

For dogs meeting the case definition, demographic data were extracted automatically from the VetCompass database, with further data relating to clinical management extracted manually from each EHR (Table 1).

**Table 1:** Definition and categorisation of demographic and clinical data extracted from the electronic health records (EHRs) of dogs with presumed DMVD attending primary-care veterinary practices in the VetCompass Programme in the UK (n = 928).

Data extracted	Definition	Categorisation
Age	Age at first record of a grade IV murmur (years)	Included as continuous variable.
Breed	Breed information entered by the participating practices was cleaned and mapped to a VetCompass breed list derived and extended from the VeNom Coding breed list [20].	All specific breeds with at least 10 dogs prescribed pimobendan or 10 dogs not prescribed pimobendan, to allow sufficient study power, with remaining dogs grouped as either ‘Purebred – Other’ or ‘Crossbred’.
Insurance status	Status at the final available EHR.	‘Insured’ or ‘Non-insured’.
Chronic comorbidity	Presence of at least one chronic comorbidity, i.e., an ongoing condition of ≥ 3 weeks duration recorded in the electronic health record at or within one month prior to grade IV murmur diagnosis and updated at approximately 3 months and 6 months.	‘Yes’ or ‘No’.
Diagnostic tests performed	At least one diagnostic test to investigate heart murmur (specifically, echocardiography, NT-pro BNP and/or radiography) recorded in the electronic health record at grade IV murmur diagnosis and updated at approximately 3 months and 6 months.	‘Yes’ or ‘No’.
Veterinary group	Individual practices in the study population are all part of a larger ‘practice group’. The practice groups were assigned a number and the group attended by each individual dog in the study recorded.	Categorised as 1–4.
Mortality	Evidence of euthanasia, unassisted death or death without the method recorded. The specific (or first listed) cause of death was recorded.	‘Died’ or ‘No evidence of death’ (with specific cause of death recorded or ‘missing’ if cause of death not available).
Congestive heart failure	Presumed or diagnosed congestive heart failure documented within the electronic health record by the attending veterinarian and/or prescription of a diuretic with concurrent clinical sign consistent with congestive heart failure documented in the electronic health record.	‘Evidence of congestive heart failure’ or ‘No evidence of congestive heart failure’.

### Target trial specification and emulation

The target trial was specified and emulated using EHR data to establish whether target trial emulation can replicate the findings of a pre-existing veterinary RCT. The primary clinical research question of interest was: ‘what is the causal effect of pimobendan prescription within 6 months of first diagnosis of a grade IV heart murmur on time to congestive heart failure?’ The causal estimands, i.e., the treatment effects of interest, were the difference in 5-year cause-specific cumulative incidence of CHF and the difference in restricted mean time lost (RMTL) to CHF. Cause-specific cumulative incidence refers to the probability of a particular event occurring over time in the presence of competing risks [22]. RMTL has been defined as the average amount of time lost due to a particular event within a specified time period and is calculated as the area under the cause-specific cumulative incidence function [23]. Therefore, in this study context, the difference in RMTL can be interpreted as the mean difference in CHF-free time in the two treatment groups, up to 5-years. In the absence of competing events, restricted mean survival time (RMST) can be calculated.

The causal contrast of interest in the current study was the intention to treat effect. For target trial emulation, it has been suggested this is referred to as an ‘intention to treat analogue’ or ‘as assigned analysis’ because the true intention may be unknown [4]. For a superiority RCT (i.e., to determine whether one treatment is superior to the comparator), the intention to treat analysis is generally used as it provides a more conservative estimate [24]. The protocol of the target trial, and the trial emulation, are summarised in Table 2.

**Table 2:** Specification and emulation of the target trial to estimate the effect of pimobendan prescription within 6 months of a grade IV heart murmur diagnosis versus no pimobendan prescription within 6 months of a grade IV heart murmur diagnosis in dogs on time to congestive heart failure, with death as a competing event.

Protocol Component	Target trial description	Emulated trial using veterinary electronic health records
Research questions	Does the prescription of pimobendan to dogs with clinical evidence of more advanced preclinical degenerative mitral valve disease (DMVD) cause a difference in (i) time to congestive heart failure (CHF), considering death as a competing event (primary outcome) and (ii) time to all-cause mortality (secondary outcome).	Same as target trial, but with advanced preclinical degenerative mitral valve disease (DMVD) defined as dogs diagnosed with first grade IV/VI heart murmur.
Eligibility criteria	Dogs aged 6 years or older with a bodyweight ≤15 kg, first grade IV/VI heart murmur recorded from January 1^st^ 2016 to December 31^st^ 2018. Dogs either prescribed pimobendan preclinically as the sole cardiovascular agent or prescribed no preclinical cardiovascular agent. Preclinical defined as no cardiac-associated clinical signs at first grade IV/VI heart murmur diagnosis and no evidence of CHF at pimobendan prescription. Exclusion criteria: Dogs with evidence of congenital cardiac disease or cardiac disease other than DMVD and dogs prescribed pimobendan or other cardiovascular agents prior to grade IV/VI murmur diagnosis. Dogs with cardiac-related clinical signs or already in CHF or prescribed furosemide at the point of first grade IV/VI heart murmur diagnosis.	Same as target trial.
Treatment strategies	1. Preclinical pimobendan prescription at first diagnosis of a grade IV/VI heart murmur. 2. No preclinical pimobendan prescription at first diagnosis of a grade IV/VI heart murmur.	1. Preclinical pimobendan prescription (as the sole cardiovascular agent) at or within 6 months of first grade IV/VI heart murmur diagnosis. 2. No preclinical pimobendan prescription (or other cardiovascular agent) at or within 6 months of first grade IV/VI heart murmur diagnosis. A 6-month grace period was specified to allow for a more realistic trial and based on discussion with a veterinary cardiologist.
Assignment procedures	Eligible dogs will be randomly assigned to either strategy at diagnosis. Owners and veterinarians involved in the dog’s care will be aware of the strategy to which they have been assigned.	Dogs are assigned to both treatment strategies at the point of murmur diagnosis, and artificially censored as soon as they are no longer compatible with this assigned treatment strategy. All observed confounding factors will be adjusted for to ensure conditional exchangeability of the groups defined by initiation of the treatment strategies.
Follow-up period	Follow up starts at enrolment (which happens when dogs are first diagnosed with a grade IV/VI heart murmur), equivalent to treatment assignment, and ends at a maximum of 5 years after.	Follow up starts at diagnosis with a grade IV/VI heart murmur and ends at a maximum of 5 years after, based on the maximum 5-year follow-up period in the EPIC trial [8].
Censoring	Loss to follow up or administrative censoring.	Same as target trial but with additional artificial censoring as soon as the dog was no longer compatible with the assigned treatment strategy.
Outcome	Primary outcome – time to CHF, with death as a competing event. Secondary outcome – time to all-cause mortality.	Same as target trial.
Causal contrasts of interest	Intention to treat effect.	Observational analogue of an intention to treat effect, meaning dogs prescribed pimobendan after 6 months continue in the ‘no pimobendan prescription’ group and dogs no longer prescribed pimobendan after 6 months continue in the ‘pimobendan prescription’ group.
Estimands	(i) Difference in 5-year cause-specific cumulative incidence of CHF and death (competing event) and difference in RMTL to CHF after 5 years. (ii) Difference in 5-year survival probabilities and difference in RMST for all-cause mortality as the outcome.	Same as target trial.
Analysis plan	Intention to treat analysis, including dogs in each treatment strategy at baseline.	Intention to treat analysis with ‘clone-and-censor’ weighting used to account for baseline confounding and immortal time bias. The artificial censoring is then addressed using inverse probability weighting. Covariate balance at the end of the grace period is assessed using standardised mean differences. Loss to follow-up beyond the grace period addressed using inverse probability of censoring weighting. A weighted non-parametric estimation of the cumulative probabilities (for the competing event analysis) and RMTL using the Aalen-Johansen approach. Dogs are followed up until artificially censored, CHF, death, loss to follow-up, or end of follow-up period, whichever came first. A weighted non-parametric Kaplan-Meier estimator will calculate the difference in survival probability and RMST for the outcome of all-cause mortality. The end point is either date of artificial censor, all-cause mortality, loss to follow-up or end of the study period (whichever came first).
Adjustment variables	Age at first diagnosis of a grade IV/VI heart murmur, breed, insurance status, chronic comorbidities, vet group and diagnostic tests performed are balanced between groups through randomisation.	Age at first diagnosis of a grade IV/VI heart murmur, breed, insurance status, chronic comorbidities, vet group and diagnostic tests performed.

### Descriptive analysis

Demographic data were described. Continuous variables were assessed graphically for their distribution and summarised using median, interquartile range (IQR) and range given most were non-normally distributed. Chi-square test was used to compare categorical variables and the Student’s t-test or Mann–Whitney U test for univariable comparison of continuous variables between two groups as appropriate [25].

### Statistical analysis of the emulated trial

For the target trial emulation to be causal, the assumptions of consistency, no interference, no unobserved confounding and positivity should hold [4]. The consistency assumption requires the exposure to be clearly and precisely defined, ensuring that each individual has a single potential outcome for each level of the exposure [26]. No interference refers to the assumption that the potential outcomes of one individual are unaffected by the treatment assignment of other individuals [27]. Based on the DAG (Fig 1), we assume that we have adjusted for enough variables to adequately control for confounding. This is a strong assumption because some confounders may remain unknown and hence unobserved. Positivity refers to the assumption that the probability of receiving each treatment conditional on measured covariates is greater than zero [4]. For the target trial emulation we consider *potential* outcomes, or what would have happened under a different treatment or exposure scenario [28,29]. Following adjustments to account for bias and achieve exchangeability between the two treatment groups, we consider ‘what if’ scenarios, such as ‘what would the risk be if all patients had been treated?’ and ‘what would the risk be if all patients had been untreated?’ [4].

The ‘clone-censor-weight’ strategy was used to account for baseline confounding and immortal time bias. Immortal time bias is possible in this study because dogs in the ‘pimobendan prescription’ group must survive from first diagnosis of a grade IV heart murmur until the pimobendan prescription date and also not develop CHF during this period to be included. To account for this, two exact copies (referred to as ‘clones’) of each dog were created in the data at baseline (the first diagnosis of a grade IV heart murmur). One clone was assigned to the ‘pimobendan prescription’ group and the other to the ‘no pimobendan prescription’ group, regardless of actual treatment. Clones were censored when their treatment no longer aligned with their assigned group. For instance, if a dog was prescribed pimobendan 3 months after baseline, the ‘no pimobendan prescription’ clone was censored at that time. This artificial censoring can introduce selection bias over time, as treatment prescription often depends on individual characteristics [30,31]. Inverse probability of censoring weighting (IPCW) was used to address this selection bias. IPCW compensates for censored patients by giving more weight to patients with similar characteristics who are not censored [32]. Probabilities of remaining uncensored at each time of event (i.e., treatment prescription, CHF and death) were predicted using a Cox regression model, including variables predictive of the artificial censoring mechanism, i.e., age, breed, insurance status, chronic comorbidities, veterinary group and diagnostic tests performed, with the artificial censoring weights calculated as the inverse of these probabilities (see S1 Appendix for further information on the clone-and-censor weight strategy) [31].

Loss to follow-up beyond the 6-month grace period was similarly addressed using IPCW, i.e., probabilities of being observed at each time of event were predicted using a Cox regression model, including the baseline variables age, breed, insurance status, chronic comorbidities, veterinary group and diagnostic tests performed, with loss to follow-up weights calculated as the inverse of these probabilities. The final weights (i.e., the artificial censoring weights and the loss to follow-up weights) were combined by multiplication to weight each clone’s contribution to the outcome model. To account for extreme weights, weights were truncated at the 1^st^ and 99^th^ percentiles [33]. Further information on the clone-censor-weight strategy is detailed in the supplementary material.

The effect of pimobendan prescription on CHF was estimated by comparing the proportion of dogs with evidence of CHF at 5-year follow-up in the ‘pimobendan prescription’ arm versus the proportion of dogs with evidence of CHF at 5-year follow-up in the ‘no pimobendan prescription’ arm. In both proportions, dogs that died before developing CHF contributed to the denominator but not the numerator [34]. The cause-specific cumulative incidence of CHF (with death as a competing event) was estimated using the Aalen-Johansen estimator, which is a generalisation of the Kaplan-Meier estimator [35].

The data required for each dog in the Aalen-Johansen analysis included the time to one of three possible outcomes (whichever came first from CHF, death, or event-free survival) and an indicator of which outcome occurred [36]. The Aalen-Johansen estimator therefore allowed for separate calculation of the cumulative incidence probabilities for the outcomes of CHF and death. The difference in RMTL to CHF between dogs prescribed pimobendan and dogs not prescribed pimobendan was calculated as the area between the cause-specific cumulative incidence curves [37]. For the survival analysis, with the secondary outcome of all-cause mortality, a weighted non-parametric Kaplan-Meier estimator was used to calculate the difference in 5-year survival probabilities. RMST was calculated as the area under the survival curve [31].

Covariate balance at the end of the 6 month grace period was assessed through calculation of the standardised mean difference (SMD). SMDs help assess whether the average value for the confounder is balanced between treatment groups [38]. For each covariate, SMDs pre- and post-clone-and-censor weighting were calculated, with SMD < 10% indicating good covariate balance between the two treatment arms [31]. The clone-and-censor weight model for all-cause mortality accounted for death and loss to follow-up within the grace period only (rather than also CHF for the competing events analysis), and so different SMDs were calculated. Biologically plausible interaction terms and transformations for the continuous covariate (age) were added to the artificial censoring and loss to follow-up weight models and their effect on the SMDs were assessed for inclusion, with interaction terms and a transformation included if they improved covariate balance [39].

Data were checked for internal validity and cleaned in Excel (Microsoft Office Excel 2013, Microsoft Corp.), with analyses conducted using R version 4.3.3 (R Core Team, Vienna, Austria). The “survival”, “boot” and “ggplot2” packages were used for the causal survival analysis and associated plots [40–42]. The ‘SDMTools’ package was used to calculate SMDs at 6 months and SMD plots were constructed in GraphPad Prism (version 8.0; GraphPad Software Inc.).

## Results

The study population consisted of 107,176 dogs with an adult bodyweight ≤ 15 kg and > 6 years of age on 1^st^ January 2016 under primary veterinary care in the VetCompass database during 2016. DMVD search terms yielded 6,012 (5.6%) candidate cases, of which 5,247 (87.3%) were manually reviewed. Of these, 928 (17.7%) met the eligibility criteria for the emulated trial. The emulated trial included 178 (19.2%) dogs prescribed pimobendan within 6 months of first grade IV murmur diagnosis and 750 (80.8%) dogs not prescribed pimobendan within 6 months of first grade IV murmur diagnosis.

### Demography of dogs in the emulated trial

Dogs prescribed pimobendan within 6 months of first grade IV murmur diagnosis had a median age of 9.9 years (IQR 8.9–11.6, range 6.0–15.9) which was younger than the median age of dogs not prescribed pimobendan (10.6 years, IQR 9.1–12.5, range 6.2–17.8) (P = 0.003). The most common breeds among cases prescribed pimobendan were the Cavalier King Charles Spaniel (33.7% of cases prescribed pimobendan; n = 60), King Charles Spaniel (6.7%; 12), Chihuahua (6.2%; 11) and Shih Tzu (5.6%; 10) along with 29 (16.3%) crossbreds. The most common breeds among cases not prescribed pimobendan were the Cavalier King Charles Spaniel (32.1%; 241), Jack Russell Terrier (10.9%; 82), Chihuahua (8.0%; 60) and Shih Tzu (6.9%; 52) along with 121 (16.1%) crossbreds (Table 3).

**Table 3:** Pimobendan prescription count (% of pimobendan cases) (n = 178) and no pimobendan prescription count (% of no pimobendan cases) (n = 750) for categorical variables recorded in dogs diagnosed with a grade IV/VI murmur attending primary-care veterinary practices in the VetCompass Programme in the UK (n = 928).

Variable	Category		Pimobendan prescription no. (%)		No Pimobendan prescription no. (%)	
Breed	Crossbred		29 (16.1)		121 (16.1)	
	Cavalier King Charles Spaniel		60 (33.7)		241 (32.1)	
	King Charles Spaniel		12 (6.7)		44 (5.9)	
	Chihuahua		11 (6.1)		60 (8.0)	
	Shih Tzu		10 (5.6)		52 (6.9)	
	Jack Russell Terrier		7 (3.9)		82 (10.9)	
	Bichon Frise		6 (3.3)		11 (1.5)	
	Yorkshire Terrier		5 (2.8)		24 (3.2)	
	Miniature Schnauzer		2 (1.1)		17 (2.3)	
	Purebred – other		36 (20.0)		98 (13.1)	
Chronic comorbidity	Yes		121 (68.0)		566 (75.5)	
	No		57 (31.7)		184 (24.5)	
Diagnostic tests performed	Yes	Echocardiography	96 (53.9)	77 (43.3)	55 (7.3)	31 (4.1)
		NT-proBNP		35 (19.7)		33 (4.4)
		Radiography		14 (7.9)		2 (0.3)
	No		82 (46.1)		695 (92.7)	
Insurance	Non – insured		106 (59.6)		588 (78.4)	
	Insured		72 (40.4)		162 (21.6)	
Veterinary Group	1		67 (37.6)		315 (42.0)	
	2		72 (40.4)		277 (36.9)	
	3		33 (18.5)		146 (19.5)	
	4		6 (3.4)		12 (1.6)	

### Emulated trial results

The final models in the emulated trial included the following covariates to generate inverse probability of artificial censoring and loss to follow-up weights: age (including a quadratic term), breed, insurance status, chronic comorbidities, diagnostic tests performed and veterinary group. Balance was improved after inclusion of an interaction term between age and chronic comorbidities in the calculation of artificial censoring weights in the competing events analysis.

### Competing events analysis

After using IPCW and checking balance was achieved (based on 6-month SMDs), pimobendan prescription led to a significant difference in 5-year cause-specific cumulative incidence of CHF (difference in cumulative incidence of −22.2%, 95% CI −30.5% to −13.6%). This equated to an adjusted 5-year cumulative incidence of CHF of 34.1% if the dogs had been prescribed pimobendan (95% CI 26.5% to 42.0%) and 56.3% if the dogs had not been prescribed pimobendan (95% CI 52.8% to 59.8%). The difference in RMTL to CHF after 5 years in dogs prescribed pimobendan compared with dogs not prescribed pimobendan was −311 days (95% CI −395 to −224 days). This indicates that dogs prescribed pimobendan within 6 months of a grade IV heart murmur diagnosis would experience 311 fewer days of health lost to CHF within a 5-year window compared to if they had not been prescribed pimobendan, when taking into account the competing risk of death.

**Fig 2:**
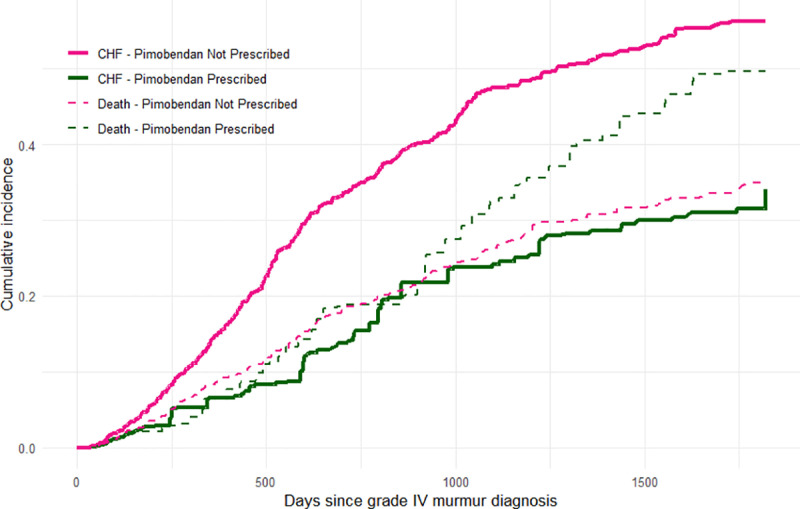
Weighted cumulative incidence, over a maximum 5-year follow-up period, estimated using the Aalen–Johansen estimator for congestive heart failure (including cumulative incidence curves for death as a competing event) if dogs attending primary-care practices in the UK were prescribed pimobendan and if dogs were not prescribed pimobendan within 6 months of first grade IV heart murmur diagnosis.

For the competing event of death (i.e., death without prior evidence of CHF), pimobendan prescription led to a significant difference in 5-year cause-specific cumulative incidence of death (difference in cumulative incidence of 14.8%, 95% CI 5.5% to 23.9%). This equated to an adjusted 5-year cumulative incidence of death of 49.7% if the dogs had been prescribed pimobendan (95% CI 40.9% to 58.3%) and 34.9% if the dogs had not been prescribed pimobendan (95% CI 31.8% to 38.0%) within 6 months of a grade IV murmur diagnosis (Fig 2).

### All-cause mortality outcome analysis

**Fig 3:**
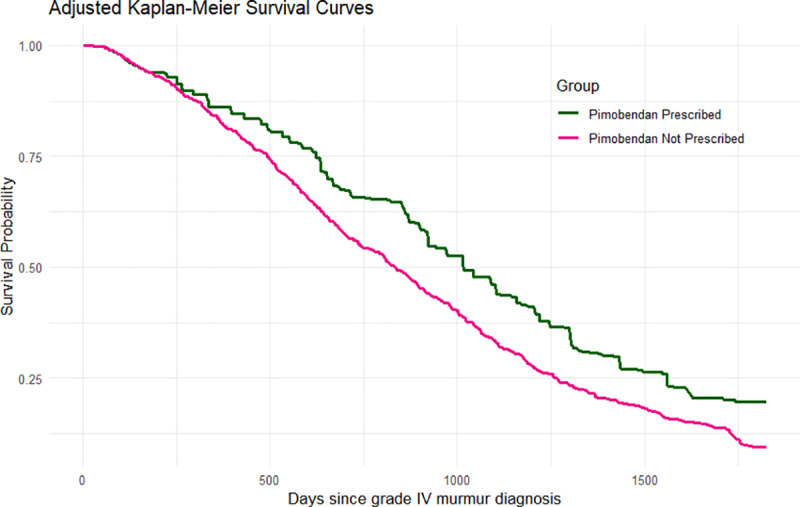
Weighted Kaplan Meier estimation (over a maximum 5-year follow-up period) of survival curves for all-cause mortality in dogs attending primary-care practices in the UK if prescribed pimobendan and if dogs not prescribed pimobendan within 6 months of first grade IV/VI heart murmur diagnosis.

When evaluating all-cause mortality as the outcome, pimobendan prescription led to a significant difference in 5-year survival probability (10.2%, 95% CI 2.3% to 17.7%). This equates to an adjusted 5-year survival probability of 19.8% (95% CI 12.5% to 26.8%) if all dogs had been prescribed pimobendan and 9.6% (95% CI 7.1% to 12.0%) if all dogs had not been prescribed pimobendan. Similarly, pimobendan prescription within 6 months of a grade IV murmur diagnosis led to a significant difference in 5-year RMST compared with no pimobendan prescription within 6 months (146 days, 95% CI 58–224 days). This equates to an adjusted 5-year RMST of 1051 days (95% CI 967–1125 days) if dogs prescribed pimobendan and 905 days (95% CI 871–940 days) if dogs not prescribed pimobendan (Fig 3).

### Model evaluation

**Fig 4:**
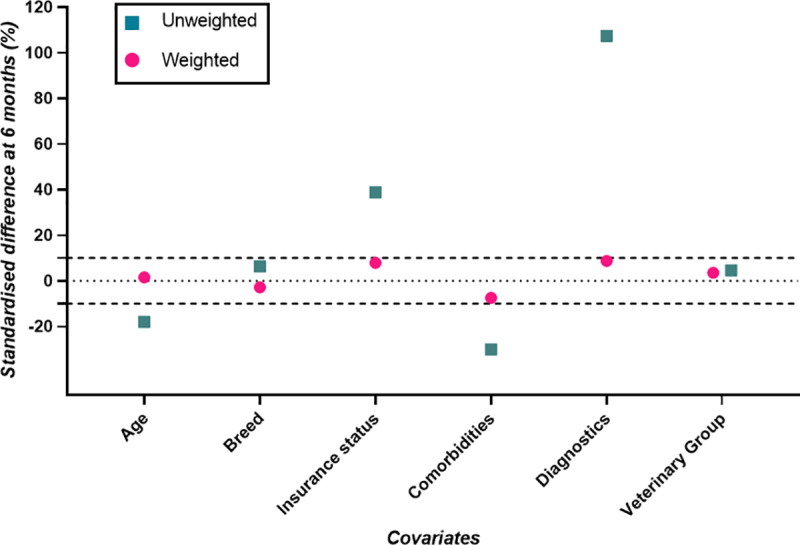
Standardised differences (%) for the outcome of congestive heart failure (with death a competing event) on the cloned data at six months after grade IV/VI murmur diagnosis, while accounting, or not, for selection bias (i.e., weighted and unweighted, respectively).

Weighted SMDs at 6 months for CHF (with death as a competing event) were all within 10%, indicating good covariate balance (Fig 4).

**Fig 5:**
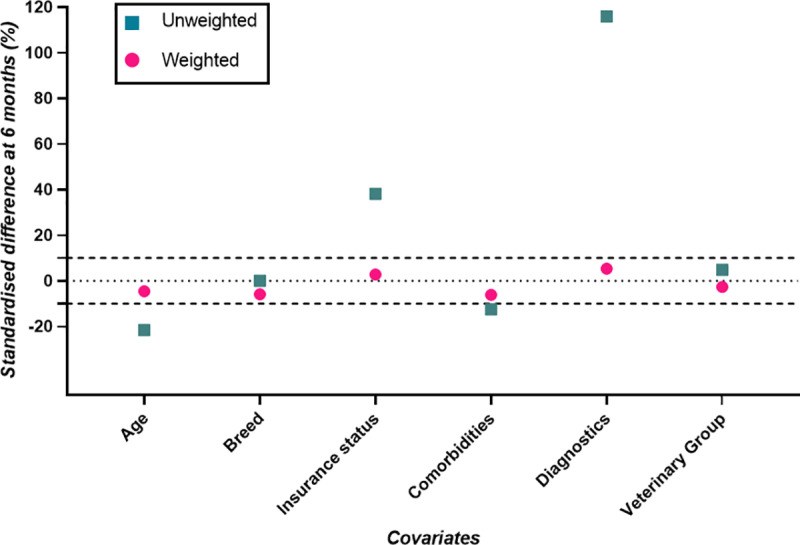
Standardised differences (%) for all-cause mortality as the outcome on the cloned data at six months after grade IV/VI murmur diagnosis, while accounting, or not, for selection bias (i.e., weighted and unweighted, respectively).

Weighted SMDs at 6 months for all-cause mortality as the outcome were all within 10%, indicating good covariate balance (Fig 5).

## Discussion

To our knowledge, the current study is the first to explore the extent to which target trial emulation approximates results from a pre-existing RCT within the veterinary literature. The results demonstrate a high degree of similarity to those found by the EPIC trial we set out to emulate. This alignment suggests that target trial emulation can serve as a robust method for deriving insights from observational data, thereby enhancing the validity of findings in veterinary research and informing clinical practice.

### CHF outcome with competing event of death

After adjustment for immortal-time bias, baseline confounders and accounting for loss to follow-up, pimobendan prescription within 6 months of a grade IV murmur diagnosis had an average causal effect of a 22.2% reduction in 5-year cumulative incidence of CHF (95% CI −30.5% to −13.6%). The 5-year cumulative incidence of CHF if all dogs had been prescribed pimobendan was 34.1% compared to 56.3% if dogs had not been prescribed pimobendan. The risk of CHF within 5 years in the EPIC trial was 33.1% in dogs treated with pimobendan and 43.2% in dogs treated with placebo [8]. Whilst the 5-year risk of CHF in dogs prescribed pimobendan is similar between the current study and the EPIC trial, there is greater disparity in the risk of CHF for dogs not prescribed pimobendan. Different case definitions, study populations and design may contribute to this difference, with the current study also accounting for the competing event of death. That said, the direction of the effect aligns between the two studies, in that prescription of pimobendan to dogs within 6 months of first grade IV heart murmur diagnosis (or at stage B2 heart disease in the EPIC trial) reduces 5-year risk of CHF.

In the current study, if all dogs were prescribed pimobendan within 6 months of first grade IV heart murmur diagnosis, an average of 311 fewer days of health were lost to CHF within a 5-year window compared to if all dogs were not prescribed pimobendan, after taking into account the competing event of death. The median time to event from treatment initiation to the composite outcome of CHF, cardiac‐related death, or euthanasia in the EPIC trial was 1228 days in the pimobendan group and 766 days in the placebo group, i.e., a difference of 462 days [8]. Whilst restricted mean time lost cannot be directly compared to median time to event, the magnitude and direction of effect is similar between the current study and the EPIC trial, albeit the benefit of pimobendan appeared greater in the EPIC trial. The current study evaluated an intention to treat estimate, rather than the per-protocol estimate measured in the EPIC trial, and accounted for the competing event of death, potentially explaining the more conservative estimate for the treatment effect in the current study.

For the competing event of death, if all dogs were prescribed pimobendan (and did not first experience CHF) the likelihood of death within 5 years without having first experienced CHF was higher compared to if all dogs were not prescribed pimobendan (51.2% vs 34.7% respectively). The median age at grade IV murmur diagnosis in dogs prescribed pimobendan in the current study was 9.9 years and 10.6 years in dogs not prescribed pimobendan. Therefore, given overall this is an old population of dogs, it is likely a large proportion would either reach CHF or die within 5 years (if follow-up were complete). However, because pimobendan prescription caused a lower 5-year cumulative incidence of CHF, it follows that there would be a greater number of dogs remaining at risk of death as a first event and therefore the 5-year cumulative incidence of death may be higher.

From the cumulative incidence curves (Fig 2), the curve for the competing event of death in dogs prescribed pimobendan appears to diverge from the curve for CHF in dogs prescribed pimobendan at approximately 3 years after grade IV murmur diagnosis. Thus, pimobendan prescription in dogs with a grade IV murmur reduces the 5-year risk of CHF, but cannot ultimately prevent dogs from dying of other causes. Sudden cardiac death was reported to be higher in dogs prescribed pimobendan (6.7%) than dogs not prescribed pimobendan (2.8%) in the EPIC trial, but not at a statistically significant level [8]. In the current study, a greater proportion of dogs died unassisted (without evidence of prior CHF) in the group *not* prescribed pimobendan (22.4%) compared to the group prescribed pimobendan (15.3%). However, it is possible some of the dogs that died unassisted did have CHF prior to death, thus the difference in sudden cardiac death between the treatment groups is more difficult to interpret based on EHRs. Notably, however, in both the EPIC trial and the current study, overall survival was longer in dogs prescribed pimobendan than in dogs not prescribed pimobendan at time zero (discussed in further detail below).

### All-cause mortality outcome

After adjustment for immortal-time bias, assessing covariate balance and accounting for loss to follow-up, pimobendan prescription within 6 months of first grade IV murmur diagnosis had an average causal effect of a 10.2% difference (95% CI 2.3% to 17.7%) in the probability of survival at 5 years. Specifically, the probability of survival at 5 years if all dogs were prescribed pimobendan was 19.8% compared to 9.6% if all dogs were not prescribed pimobendan, i.e., the 5-year survival probability approximately doubled if all dogs were prescribed pimobendan compared to if all dogs were not prescribed pimobendan. As mentioned above, given the median age of dogs in the study, it is unsurprising the majority of dogs died within 5 years. However, a significant increase in survival if all dogs were prescribed pimobendan is still evident at 5 years.

Within the 5-year follow-up period, the mean survival time was 1051 days if all dogs were prescribed pimobendan within 6 months of first grade IV heart murmur, compared with 905 days if all dogs were not prescribed pimobendan. These findings align very closely with the intention to treat estimate of median survival time of 1059 days in the pimobendan group and 902 days in the placebo group in the EPIC trial [8]. All cause mortality as an outcome is straightforward to measure and less prone to bias and subjectivity than other outcomes in studies confirming the safety of treatment. A robust beneficial treatment effect was evident in the current study, as seen in the original EPIC study. Although adaptations were made in the current study for a primary-care population, the similarity in results between the current target trial emulation and the previously published RCT supports the treatment effect and provides strong evidence supporting the use of observational data to emulate veterinary RCTs.

### Overall discussion and limitations

The outcomes of CHF (with a competing event of death) and all-cause mortality were evaluated in the current study. Prescription of pimobendan to dogs within 6 months of first grade IV murmur caused approximately 10 months fewer of health lost to CHF within a 5-year window and an increase in survival of approximately 5 months. These findings suggest that pimobendan prescription within 6 months of first grade IV heart murmur diagnosis has a greater effect in delaying CHF, rather than prolonging survival, although there is still an overall survival benefit.

First grade IV heart murmur diagnosis was set as time zero, compared with stage B2 DMVD in the EPIC trial [8]. It has been reported that 48.7% dogs in stage B2 DMVD have a grade IV murmur [10], therefore it is likely the dogs in the current study represent different ACVIM disease stages. However, the eligibility criteria in the current study were designed to exclude dogs already diagnosed with clinical signs of CHF, i.e., stage C or D DMVD at the point of grade IV murmur diagnosis. Therefore, the dogs in the current study should represent dogs in Stage B1 or B2 DMVD. Stage B2 DMVD was proposed as the intervention point for pimobendan treatment following the EPIC trial results [8], but there could be other DMVD populations that could benefit from pimobendan prescription. Based on the current study findings, dogs with a grade IV murmur might represent such a population. There were 43.3% of dogs prescribed pimobendan that underwent echocardiography in the current study. Therefore, it is likely that at least this proportion of dogs represented stage B2 DMVD as this is the only preclinical stage currently recommended for treatment. Thus, it could be that most dogs in the current study represented stage B2 DMVD, or alternatively there could be a similar treatment benefit in dogs with stage B1 DMVD that have a grade IV heart murmur. The findings of this study could inform a further RCT or pragmatic clinical trial including all dogs in stage B DMVD with a grade IV heart murmur. Given that full cardiac diagnostic work-up might not be accessible or preferred by all owners of dogs with a heart murmur, preclinical grade IV murmur diagnosis may represent a suitable stage to initiate pimobendan treatment in primary-care practice.

The limitations of this study are largely based on the nature of retrospective analysis of EHR data, including issues related to unobserved confounding, missing and misclassified data and application of a case definition to the data available [43]. Causal language was used throughout this study, based on the causal assumptions of consistency, no interference, no unobserved confounding and positivity. Although expert opinion was sought in construction of the DAG, it is possible unmeasured confounders could influence the effect estimates calculated. For example, heart size has been reported as a confounder [8,44]. Other variables collected in the study might in part encapsulate heart size, such as diagnostic tests performed, but this is difficult to fully quantify. Nonetheless the agreement between the current study findings and the EPIC trial are reassuring and suggest that major confounders have been accounted for.

## Conclusions

This study provides *proof of concept* that target trial emulation can replicate results from an existing RCT in veterinary research, with similar treatment effects observed for pimobendan across both studies. The consistency of these findings supports the greater use of target trial emulation within veterinary research, particularly when RCTs are not practical, ethical or are too costly. Clinically, prescription of pimobendan to dogs within 6 months of first grade IV heart murmur caused (i) a 22% lower risk of CHF at 5 years and approximately 10 months fewer of health lost to CHF, and (ii) A 10% increase in the probability of surviving at 5 years and a 5-month survival benefit. This suggests preclinical grade IV murmur diagnosis may be an appropriate intervention stage for pimobendan prescription in dogs with presumed DMVD, which further studies might help to clarify.

## Supporting information

S1 Appendix Clone-and-censor weight strategy. Further information.
(DOCX)

S1 Fig Possible patient scenarios that could occur within the 6-month grace period and how these would be addressed with the clone-and-censor approach. (TIF)

## Abbreviations

ACVIM: The American College of Veterinary Internal Medicine
CHF: congestive heart failure
CI: confidence interval
DAG: directed acyclic graph
DMVD: degenerative mitral valve disease
EHR: electronic health record
EPIC: Effect of Pimobendan in Dogs with Preclinical Myxomatous Mitral Valve Disease and Cardiomegaly
IPCW: inverse probability of censoring weighting
IQR: interquartile range
RCT: randomised controlled trial
RMST: restricted mean survival time
RMTL: restricted mean time lost
SMD: standardised mean difference
