# Supplementary material for: Emulating the EPIC trial using VetCompass primary-care data: causal effects of pimobendan in UK dogs with grade IV/VI heart murmurs
Source: PLoS One. 2025 Jun 18;20(6):e0325695. doi: 10.1371/journal.pone.0325695 (PMC12176212; doi:10.1371/journal.pone.0325695)
Supplement: S1 Appendix — Further information. (DOCX) [file pone.0325695.s001.docx]

S1 Appendix. Clone-and-censor weight strategy. Further information.

Fig 1 (based on Maringe et al. 2020) illustrates possible scenarios that could occur within the 6-month grace period and how these scenarios would be addressed using the clone-and-censor approach.

Fig 1: Possible patient scenarios that could occur within the 6-month grace period and how these would be addressed with the clone-and-censor approach.

Dog A is prescribed pimobendan within the 6-month grace period, therefore the ‘pimobendan prescription’ clone is *not* censored, but the ‘no pimobendan prescription’ clone *is* censored at the date of prescription. Dog A contributes to the artificial censoring weight models in both treatment arms until the date of prescription. If dog A reaches CHF, dies or is lost to follow-up after pimobendan prescription, the event and follow-up time only contribute to the ‘pimobendan prescription’ arm in the outcome model. Dog B is not prescribed pimobendan within the grace period and so the ‘no pimobendan prescription’ clone is *not* censored, whereas the ‘pimobendan prescription’ clone *is* censored at 6 months. Therefore, dog B contributes the full 6 months to the weight models. If dog B reaches CHF, dies or is lost to follow-up beyond the grace period, the event and follow-up time only contribute to the ‘no pimobendan prescription’ arm in the outcome model. Dog B could be prescribed pimobendan after the grace period, but as this is an ITT analysis, the dog would still be compatible with the ‘no pimobendan prescription within 6 months’ strategy. Dog C is still compatible with both the ‘pimobendan prescription’ and ‘no pimobendan prescription’ arms at the point of CHF, death or loss to follow-up. Therefore, this dog is not censored in the weight models and rather contributes to both treatment groups equally in the weight and outcome models.
