# Supplementary figures and images for: Emulating the EPIC trial using VetCompass primary-care data: causal effects of pimobendan in UK dogs with grade IV/VI heart murmurs
Source: PLoS One. 2025 Jun 18;20(6):e0325695. doi: 10.1371/journal.pone.0325695 (PMC12176212; doi:10.1371/journal.pone.0325695)

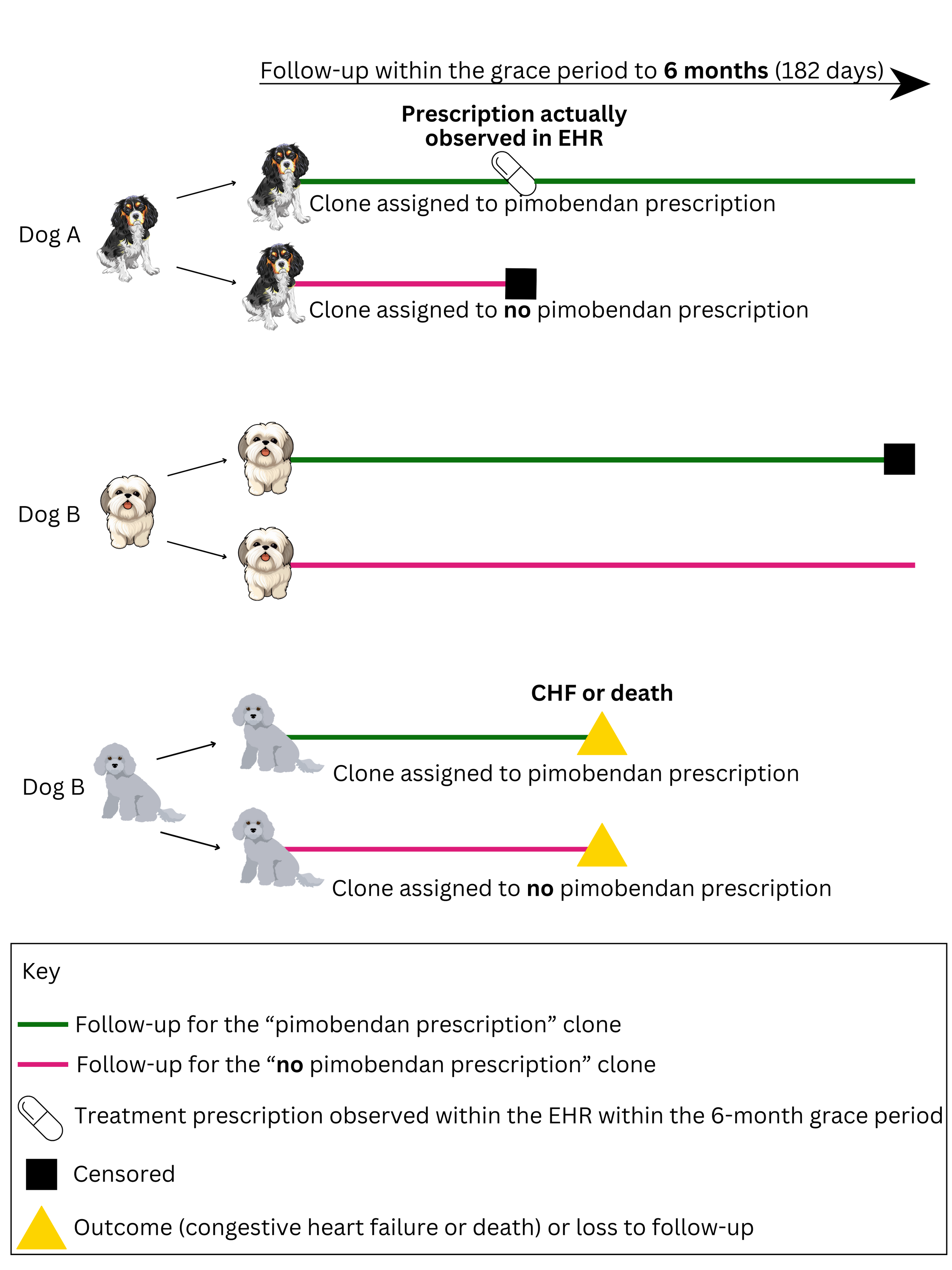

Supplement: S1 Fig — (TIF) [file pone.0325695.s002.tif]
